# The perspectives of clients and unqualified allopathic practitioners on the management of delivery care in urban slums, Dhaka, Bangladesh - a mixed method study

**DOI:** 10.1186/1471-2393-10-50

**Published:** 2010-09-07

**Authors:** Tasnuva Wahed, Allisyn C Moran, Mohammad Iqbal

**Affiliations:** 1 Reproductive Health Unit, Public Health Sciences Division, ICDDR, B, GPO Box 128, Dhaka 1000, Bangladesh; 2 Department of International Health, Johns Hopkins Bloomberg School of Public Health, Baltimore, MD, USA; 3 Social and Behavioural Sciences Unit, Public Health Sciences Division, ICDDR, B, GPO Box 128, Dhaka 1000, Bangladesh

## Abstract

**Background:**

BRAC is implementing a program to improve maternal and newborn health among the urban poor in the slums of Bangladesh (Mansohi), funded by the Bill & Melinda Gates Foundation. Formative research has demonstrated that unqualified allopathic practitioners (UAPs) are commonly assisting home-delivery. The objective of this study was to explore the role of unqualified allopathic practitioners during home delivery in urban slums of Dhaka.

**Methods:**

This cross-sectional study was conducted between September 2008 and June 2009 in Kamrangirchar slum in Dhaka, Bangladesh, using both qualitative and quantitative research methods. Through a door-to-door household survey, quantitative data were collected from 463 women with a home birth and/or trial of labor at home. We also conducted seven in-depth interviews with the UAPs to explore their practices.

**Results:**

About one-third (32%) of the 463 women interviewed sought delivery care from a UAP. We did not find an association between socio-demographic characteristics and care-seeking from a UAP, except for education of women. Compared to women with three or more pregnancies, the highest odds ratio was found in the primi-gravidity group [odds ratio (OR): 3.46; 95% confidence interval (CI): 1.65-7.25)], followed by women with two pregnancies (OR: 2.54; 95% CI: 1.36-4.77) to use a UAP. Of women who reported at least one delivery-related complication, 45.2% received care from the UAPs. Of 149 cases where the UAP was involved with delivery care, 133 (89.3%) received medicine to start or increase labor with only 6% (9 of 149) referred by a UAP to any health facility. The qualitative findings showed that UAPs provided a variety of medicines to manage excessive bleeding immediately after childbirth.

**Conclusion:**

There is demand among slum women for delivery-related care from UAPs during home births in Bangladesh. Some UAPs' practices are contrary to current World Health Organization recommendations and could be harmful. Programs need to develop interventions to address these practices to improve perinatal care outcomes.

## Background

There is a serious shortage of human resources to provide healthcare services in the South Asian region. This shortage is exacerbated by an inequitable distribution of providers by geographic area (rural versus urban), skill-mix (nurses/midwives versus specialists), level of health institution (primary versus tertiary), gender (male versus female) and coverage of services [1-3]. As a result, informal healthcare providers, predominantly unqualified allopathic practitioners (UAPs), provide a majority of healthcare for poor households in India, Pakistan, and Bangladesh [4-8]. These providers are popular because of low cost, easy access, convenient hours, willingness to make house calls, and their acceptance by the community [9,10]. UAPs are generally either untrained or have limited training in specific disease areas such as diarrhea, malaria, acute respiratory infection, Integrated Management of Childhood Illness, AIDS, tuberculosis, and safe motherhood. In Bangladesh and India, they are commonly referred to as rural medical practitioners, medicine-sellers, injectionists, injection doctors, needle men, quacks, *pallichikitshaks*, or village doctors [11-14].

In Bangladesh, the demand for and supply of UAPs is growing rapidly. Cockcroft *et al. *found that the proportion of outpatient visits conducted by unqualified practitioners rose from 52% in 2000 to 60% in 2004 [15]. In 2004, there were 110,000 UAPs in Bangladesh [16], while in 2007, there were 12 village doctors and 11 drug sellers per 10,000 population [17]. Along with the increased number of UAPs, their areas of practice are also expanding. Results of recent studies have showed that UAPs provide treatment for simple to more serious conditions, ranging from diarrhea and fever to reproductive health and maternity care. In a rural area of Bangladesh, almost half of the women in the study reported receiving treatment from UAPs for complications of pregnancy, delivery and the postpartum [13]. Another study in the urban slums of Bangladesh demonstrated that families preferred seeking care from unqualified allopathic practitioners for perceived postpartum complications compared to other sources [18]. UAPs have poor knowledge about pregnancy-related complications [19]; however, they are the preferred providers for complications during home delivery. UAPs also manage postpartum complications. One study found that UAPs incorrectly managed postpartum bleeding with ergometrine. They also play an important role in referring women to health facilities, even when traditional birth attendants (TBAs) prefer to manage the complications at home [12,20].

In Bangladesh, the majority (85%) of births take place at home [21]. Although there is some evidence of UAP's management of pregnancy and postpartum complications during home births, little is known about their role during labor and delivery or the determinants of seeking treatment from this type of provider. Urbanization is occurring at a rapid pace in Bangladesh (3.5% per year), with the majority of growth in poor urban slum areas [22]. Women in urban slums are more likely to give birth at home than urban non-slum women (88% versus 54% respectively) [23]. Research has also shown that care-seeking from UAPs is more prevalent among disadvantaged populations [7]. Therefore, it is essential to assess the factors that influence the use of UAPs and their practices in urban slums.

Bangladesh Rural Advancement Committee (BRAC) is implementing a project to improve maternal, newborn, and child morbidity and mortality in urban slums in Bangladesh, funded by the Bill & Melinda Gates Foundation [24]. Preliminary qualitative findings from formative research have documented significant UAP involvement during home births, including injections and intravenous saline infusions [25]. The objective of this paper is to explore care-seeking from UAPs during labor and delivery and delivery care practices in urban slums in Dhaka, Bangladesh. The findings will be used to inform the Manoshi program to improve interventions and minimize potentially harmful practices.

## Methods

### Study design and settings

Data for this cross-sectional study were collected between September 2008 and June 2009 in Kamrangir Char slum in Dhaka, Bangladesh, using both qualitative and quantitative research methods. BRAC's Manoshi program has maintained an office in Kamrangir Char since March 2007, and divided the slum into seven segments. According to the Manoshi Baseline Survey (2007), about 85,557 people were living in the study area.

### Study participants

A door-to-door household survey was conducted to explore health care-seeking behaviors of slum women from UAPs during delivery. The study population for the survey included all women of reproductive age (15-49 years), with a trial of labor and/or birth at home within six months preceding of the survey. Women who died during delivery or within 42 days of giving birth were excluded. Maternal death was a rare event, and we excluded maternal deaths due to the sensitive nature of the information surrounding those deaths. In addition, a concurrent study on medical and social causes of death was conducted in the same study area, and we did not want to over-burden respondents [26].

In-depth qualitative interviews with UAPs were also conducted to explore their delivery-care practices. The UAPs were mainly medicine-sellers who worked in pharmacies in or near the study area.

### Sample size, data sources and measurement

The study was designed to measure use of oxytocin to augment labor during home births. Since there were no data available on the proportion of women who receive oxytocin during labour at home; an estimate of 50% was used to calculate the sample size (n = 384). Considering a non-response rate of 20%, a total of 480 women were required.

The subjects were randomly selected for the quantitative survey. Since there was no household listing of women with recent deliveries, the centre point of each area was identified through informal discussions with Manoshi's service providers and with key community people. A bottle was spun in the centre point of each area, and based on the direction of the bottle, trained male and female interviewers visited every household until the sample was achieved. In households where eligible women were available, a structured interview was administered after the woman gave written consent. The interviewers cross-checked each completed questionnaire. A research officer also monitored the data-collection process through regular field visits.

In-depth interviews were carried out using a triad approach, identified using purposive sampling. Each triad consisted of one woman who reported using oxytocin to augment labor, the TBA who conducted that woman's delivery, and the UAP who provided medicine/treatment. We purposively selected women who reported using oxytocin during delivery but who did not participate in the quantitative survey. Then we asked her to identify the TBA and the UAP who conducted the delivery. Interviews with TBAs and UAPs were conducted at their home and pharmacies respectively. A total of 21 in-depth interviews were conducted (UAP = 7, TBA = 7, and women = 7) from the seven areas (n = 21). As the in-depth interviews with women and TBAs mainly focused on the use of oxytocin, we present findings of the in-depth interviews with seven UAPs. On an average, each interview took two hours, and all but one interview was audio-recorded. One UAP did not permit audio-recording, so his interview was documented by a note-taker.

### Variables

A quantitative structured questionnaire was developed which included socio-demographic, reproductive characteristics, and delivery care-seeking variables. This questionnaire was pretested in other urban slums, excluding the study area. The socio-demographic variables included women's age, education, and average monthly family income (in taka). Because of low literacy rate of slum women, we categorized women's education into two groups based on the completed years of schooling (none, any education). The reproductive variables included gravidity (number of total pregnancies including last birth), current number of living children, and knowledge of at least one of five danger signs during pregnancy (severe headache, high fever, blurry vision, convulsion, and vaginal bleeding [27]).

We collected information on the reported use of medicines and treatments at home during the last childbirth. Women were also asked if they had experienced any type of delivery-related complications, such as labor pain for more than 12 hours, excessive bleeding, high fever, convulsion, baby's hand and feet came first during delivery, perineal tear, retained placenta, and ruptured uterus.

We defined the dependent variable as whether the woman received any care (medicine/treatment) from a UAP for delivery and the immediate postpartum during home-delivery or if she was referred by the UAP to any health facility.

The in-depth interview guideline included a variety of themes, including the type of medicine and treatment provided by the UAP during the three stages of labor (to start or induce labor, to augment labor, and to remove the placenta), during the immediate postpartum (management of excessive bleeding), in addition to referral practices.

### Data analysis

#### Quantitative data

We checked and edited data for accuracy, consistency, and completeness. We used Oracle 10 g software for data entry and the SPSS software (version 11.5) for analysis. The associations between the demographic and reproductive variables of women and the use of UAPs during delivery were examined using Pearson's chi-square test. We also generated three models using univariate and multivariate logistic regression to measure unadjusted (Model 1), partially adjusted (Model 2), and adjusted odd ratios (Model 3) to identify the predictors of using a UAP. Model 2 was generated in two steps. We first included only socio-demographic characteristics (women's age group, education, and monthly family income). In the second step, we included only reproductive characteristics, such as gravidity, knowledge of danger-signs, and reported delivery-related complications. We included all the socio-demographic and reproductive variables in the final model. As we were interested to test seven predictors, we needed 106 cases (50+ 8 k, where k is the number of independent variables) for the overall model and 139 cases (104+5 k) for the individual predictors. We did not compare clusters; so, we did not take any measures to adjust for clustering errors. We considered dichotomous responses of using or not using a UAP during delivery as the outcome variable [28,29].

#### Qualitative data

Each in-depth interview was transcribed in Bangla (Bengali). We prepared a format containing themes and sub-themes based on the pre-defined issues included in the in-depth interview's guideline. A summary of each interview in English was then developed using this format. These sub-themes were compiled [30]. The analysis was completed manually, and no qualitative analysis software was used.

### Ethical issues

Ethical approval was obtained from the ICDDR, B Research and Ethical Review Committees. All participants gave written consent.

## Results

### Quantitative findings

#### Health care-seeking during delivery

Of the 480 women with a recent delivery, 463 (96.5%) were successfully interviewed. All 463 women had a trial of labor at home, with 87.7% of women giving birth at home. The remainder (12.3%) gave birth in a health facility. Among women with a birth at home or trial of labor at home, almost all reported that a TBA assisted with the delivery. About half of women (51.2%) also reported that a family member, relative, neighbor or no one were present with the birth. Only one woman reported that a nurse (skilled provider) assisted during the home delivery. In addition, women reported seeking treatment from spiritual healers/*kabiraj*/homeo-doctors (10.2%). Of the 463 women, 149 (32.2%) received medicine, and/or treatment and/or were referred by the UAPs to any health facility (data not shown).

#### Socio-demographic characteristics

The socio-demographic characteristics of the study women are presented in Table 1. Although the majority (63%) of the respondents were aged 20-29 years, younger women were more likely to use a UAP for delivery care than older women (p < 0.05). Women with any education (38.5%) were also more likely to report delivery services from a UAP compared to women with no education (21.0%) (p < 0.05).

**Table 1:** Socio-demographic and obstetric characteristics of women by their use of unqualified allopathic practitioners (UAPs) during labor and delivery

Characteristic	n	% of women who used UAPs during delivery	p value	Pearson Chi-square
**Age group (years)**				
15-19	69	40.6	p = 0.037	8.482
20-24	171	35.7		
25-29	121	31.4		
≥ 30	102	21.6		
**Education**				
None	167	21.0	p < 0.001	15.076
Any education	296	38.5		
**Average monthly family income (taka)**				
≤ 5000 (≤ 71 US$)	157	32.2	p = 0.346	2.123
5001-8000 (71.1-114 US$)	154	31.0		
≥ 8001 (≥ 114.1 US$)	152	28.0		
**Gravidity (number of total pregnancies)**				
1	115	46.1	p < 0.001	24.975
2	133	38.3		
≥ 3	215	20.9		
**Number of living children**				
0-1	152	44.1	p < 0.001	21.640
2	130	34.6		
≥ 3	181	20.4		
**Knowledge on any danger-sign during pregnancy**				
Yes	255	36.1	p = 0.029	3.950
No	208	27.4		
**Reported any delivery-related complications**				
Yes	197	45.2	p < 0.001	26.537
No	266	22.6		
**Total**	**463**	**32.2**		

Although there was no significant association between the monthly family income of the respondents and the use of UAPs during delivery, 67.2% (311 cases) of respondents had an average monthly income up to Tk 8,000 (US$ 114) (Table 1).

#### Type of delivery care practice

Of the 149 cases where care was sought from a UAP, treatment to start or increase labor pain was most common (133 cases, 89%). Only a few women sought treatment during the third stage of labor (after the baby was born but before removal of placenta) (5 cases) or for excessive immediate postpartum bleeding (8 cases). Only 6% (9 cases) were referred by a UAP to any health facility (data not shown).

#### Reproductive characteristics

The reproductive characteristics are presented in Table 1. We found a strong association (p < 0.001) between gravidity and number of living children and care-seeking from a UAP during delivery respectively. Women with fewer pregnancies (primi-gravidity 46.1% and second gravidity 38.3%) were more frequent users of UAPs. Similarly, women who had fewer living children were more likely to seek treatment from the UAPs. Moreover, the findings showed a significant association between women's knowledge of pregnancy danger-signs and care-seeking from a UAP. Of the 463 women, 197 (42.5%) reported at least one delivery-related complication. Of them (n = 197), 45.2% reported seeking care from a UAP compared to 22.6% of 266 women who did not report any complication (p < 0.001) (Table 1).

### Predictors of using UAPs during delivery

Table 2 presents the results of logistic regression. Although gravidity and the number of living children were significantly associated with the use of UAP, they were highly correlated (r = 0.893, p < 0.01). So, we only included gravidity in the final model. Similarly, women's education and knowledge of danger-signs were significantly correlated (r = 0.296, p < 0.01); so, we used women's education in the final model (Model 3).

**Table 2:** Unadjusted, partially adjusted, and adjusted odds ratios of use of UAPs during labor and delivery among women in KamrangirChar slum in Dhaka, 2008

Characteristic	Model 1 Unadjusted OR (95% CI)	Model 2 Partially Adjusted OR (95% CI)		Model 3 Adjusted OR^c^ (95% CI)
		OR^a^	OR^b^	
**Socio-demographic**				
**Age group**				
15-19	2.48 (1.27-4.87)	1.77 (0.87-3.60)		0.61 (0.23-1.60)
20-24	2.01 (1.14-3.56)	1.53 (0.847-2.79)		0.71 (0.32-1.57)
25-29	1.67 (0.91-3.06)	1.49 (0.801-2.78)		1.18 (0.60-2.34)
≥ 30	1.00	1.00		1.00
**Woman's education**				
Any education	2.36 (1.52-3.66)	2.04 (1.27-3.28)		1.57 (0.96-2.58)
None	1.00	1.00		1.00
**Monthly family Income (in taka)**				
≤ 5000 (≤ 71 US$)	0.89 (0.56-1.44)	0.95 (0.58-1.55)		1.04 (0.63-1.74)
5001-8000 (71.1-114 US$)	0.70 (0.43-1.14)	0.825 (0.49-1.36)		0.882 (0.52-1.49)
≥ 8001(≥ 114.1 US$)	1.00	1.00		1.00
**Reproductive**				
Gravidity				
1	3.23 (1.97-5.28)		2.79 (1.68-4.65)	3.46 (1.65-7.25)
2	2.35 (1.45-3.79)		2.23 (1.35-3.67)	2.54 (1.36-4.77)
≥ 3	1.00		1.00	1.00
**Knowledge on any danger-sign during pregnancy**				
Yes	1.49 (1.01-2.23)		1.42 (0.93-2.17)	-
No	1.00		1.00	
**Reported any delivery-related complication**				
Yes	2.82 (1.89-4.22)		2.66 (1.75-4.02)	2.56(1.68-3.90)
No	1.00		1.00	1.00

a: Adjusted for socio-demographic variables (age group, woman's education, monthly family income) b: Adjusted for reproductive variables (gravidity, knowledge on any danger-sign during pregnancy, reported any delivery-related complication)

c: Adjusted for socio-demographic and reproductive variables (age group, woman's education, monthly family income, gravidity, reported any delivery-related complication)

The findings showed that women's age-group, education, gravidity, knowledge of danger-signs, and reported delivery-related complications were significantly associated with the use of UAPs (Model 1). Partially adjusted analysis (Model-2) was done to assess possible contribution to the model by the socio-demographic or reproductive characteristics separately. Apart from Model 2, when only the socio-demographic variables were included, women with any education were two times more likely to use UAPs compared to women with no education. When we included only reproductive variables, gravidity and delivery-related complications were found to be significant factors.

However, in the full model, when all the variables were adjusted (Model 3), gravidity and reported delivery-related complications were significant. Compared to women with three or more pregnancies, the odds ratio (OR) was the highest in the primi-gravidity group (OR: 3.46; 95% CI: 1.65-7.25), followed by women with two pregnancies (OR: 2.54; 95% CI: 1.36-4.77). Women who reported any complication during delivery were 2.5 times more likely to use a UAP compared to those who did not report any complication (OR: 2.56; 95% CI: 1.68-3.90).

### Qualitative findings

#### Management of first or second stage of labor

In most cases, the UAPs reported that a '*dai*' (Traditional birth attendant) would notify them if a woman experienced problems with the delivery. Usually, a family member was sent to the UAP's shop to bring him to the house where the delivery was taking place. All UAPs reported providing the syntocinon group of medicines (e.g. piton-s, oxin) to augment labour pain and facilitate delivery at home; *"To increase pain if there is no pain, I try with the injection of oxytocin group or slow drip of saline" (a UAP of Muslimbag slum) (ID-223)*. The majority of UAPs stated that they gave this type of medication through IV saline. Almost every respondent reported that they also added vitamins to the saline infusion (vitamin B-complex, vitamin C). One UAP mentioned mixing only vitamins in the saline to give the women strength if she was weak.

*"If a saline (one B-50 injection which is vitamin B-complex) is pushed for weakness, it will rehydrate the woman or if an injection (piton-s) is pushed, it will increase pain. Piton-s or Linda-s injection is mixed in saline. Piton is old name but the present name is Linda-s" (a UAP of Muslimbag slum, age 28 years, 12 years of schooling, attended 3-month training on primary treatment, and duration of involvement in this profession is 11 years) (ID-223)*.

#### Management of third stage of labor

Right after the delivery of baby, two of seven UAPs disclosed that they provided the syntocinon group of medicine (oxytocin) to facilitate delivery of the placenta.

"It may be six months ago, the delivery was completed but there was a retained placenta. The saline was continued. I gave another injection (1 ampoule piton-s) in that saline and increased the saline drops to make powerful. After that, the placenta was removed." (a UAP from Mominbag slum, age 54 years, 12 years of schooling, attended 6 - month training, duration of involvement in this profession is 9 years) (ID-422)

#### Management of excessive bleeding just after childbirth

Most UAPs had some experience in managing excessive bleeding. They provided oral allopathic medicines, such as oral anaroxyl (generic name: adrenochrome monosemicarbazone), or oral methergine (generic name: ergometrine maleate), and injections (anaroxyl injection or methergine injection) to manage excessive bleeding. They also pushed intravenous injections. Some UAPs had a relationship with qualified allopathic healthcare providers as these doctors often worked from the drug shops in the afternoons or after office hours. If a woman had the ability to provide a fee to a qualified doctor, these UAPs called them for home service.

"I give anaroxyl injection or methergin injection for excessive bleeding after delivery. I also provide oral tablets, such as methergine tablet, anaroxyl tablet with antibiotic (amoxicillin group of medicine: fymoxyl capsule, moxacil capsule, tycil capsule) according to the need. Suppose, I give one or two or three ampoules of anaroxyl injection according to the need. This injection is given directly into vein. As the Gynae doctor visits my shop each day, I have an advantage. If I see that bleeding could not be managed after providing treatment, I call her and then she guide me what to do. If any one is able to provide her a fee (Tk 200/US$ 2.8, if woman's house is far, then (Tk 300/US$ 4.3), I bring her to that house." (a UAP from Mominbag slum) (ID-422)

Two UAPs also cited that they provided oxytocin injection along with methergine injection to manage excessive bleeding.

"I attended a serious case. The woman had excessive bleeding after delivery. They (family members) came to me 3 o'clock at night. I went and found that the woman kept lying at home. I referred her to a health facility. But they were like village people and started requesting me. I told them that, if it was not managed (if there is uncontrolled bleeding), I have nothing to do. Then I gave two oxytocin injections in a 1000 ml saline bag and one methergine injection through canula. She was managed by God." (a UAP from Alinagar slum, age 50 years, 8 years of schooling, attended 1- month training on primary treatment, and duration of involvement in this profession is 24 years) (ID-626)

#### Referral care

##### Why refer

Five of the seven UAPs reported that they referred a woman if the delivery was not completed within 30 minutes to three hours after initiating a saline drip and/or oxytocin or if the woman had a retained placenta.

"Placenta is removed within 10-15 minutes after birth. If it is not happened by this time by God, I refer. Patient goes according to their own choice. If I refer, I send the patient to Azimpur Maternity, Dhaka Medical College Hospital or Mitford Medical College Hospital because a very few people afford the cost of services at a private hospital." (a UAP from Koilar Ghat slum, age 35 years, 14 years of schooling, attended 6-month training on primary treatment, and duration of involvement in this profession is 18 years) (ID-324)

Two of the seven UAPs reported that they referred the woman to a health facility if the baby's head was large, expected date of delivery (EDD) had passed but there was no labor pain and cervix was not opened, or if *dai *(TBA) reported that the baby was breech or other abnormal position.

Two UAPs reported referring women to a facility if their blood pressure (BP) was high or more than 150 (mm of Hg), as they feared that it might lead to convulsion.

A few respondents stated that they referred women over 30 years of age, those with a birth interval of more than five years, if the woman's pulse rate was high, or if she had blurry vision.

"If I find high BP, I do not manage because it may cause convulsion. I refer it. I consider the age of the last delivered child because if age is more than five years, the possibility of normal delivery is decreased among many cases. I do not handle these cases. I refer them. It is very unusual to conduct normal delivery if the woman's age is 30 years or more. So, I refer them to Dhaka Medical College Hospital or other health centers" (a UAP from Nabinagar slum, age 38 years, 16 years of schooling, attended 6-month training on primary treatment, duration of involvement in this profession is 11 years) (ID-727)

##### Where refer to

Most respondents cited that they referred women to the government hospital, as services are provided free or at low cost. However, one provider commented that he needed to ensure that the woman had appropriate paperwork to be admitted to one government specialist hospital.

"I refer woman to the maternity hospital but it is not possible for all to go to the hospital. The Maternity Hospital does not allow if any one does not have a card. I also send women to the Dhaka Medical College Hospital, Sohrawardi Hospital, Mitford Medical College Hospital (government facility)". (a UAP of Muslimbag slum) (ID-223)

The findings of in-depth interviews also showed that some UAPs sent women to the private-for-profit hospital if they could pay for the care.

"I referred a delivery case after giving two doses of injection. I have to refer her because it was her first pregnancy, and the head of the baby was large. So, it could not be delivered at home. I sent her to Care View Clinic, Rayer Bazar. Then the doctor (MBBS) conducted normal delivery with episiotomy. Both mother and baby were fine. Sometime I refer the poor people to Dhaka Medical College Hospital and Mitford Medical College Hospital as they cannot afford to pay for the services of a private clinic" (a UAP of Alinagar slum) (ID-626)

## Discussion

Seeking healthcare from informal healthcare providers during delivery is very common in South Asia [31-34]. However, few studies in Bangladesh have explored the role of UAPs in managing childbirth. The present study explored clients' and UAPs' points of view on the management of delivery care in urban slums using a mixed method approach. The findings of the study showed that 32% of the study participants sought delivery care from the UAPs. We did not find an association between socio-demographic characteristics and health care-seeking from a UAP during delivery, except for education of women. Women with any education were more likely to seek delivery care from UAPs, which differed from Ahmed's study findings. It should, however, be noted that Ahmed's study was conducted in rural areas of Bangladesh and healthcare seeking was not limited to delivery-related complications [7]. Another study by Moran, Wahed & Afsana in slum areas of Bangladesh and a few studies in rural India showed that women with any education were more likely to use oxytocin to augment labor administered by UAPs at home [35,36].

This study found a strong association among women with fewer pregnancies and fewer living children and health care-seeking from a UAP during delivery. Primi-gravidity was significantly associated with the use of oxytocin from unskilled practitioners during home-deliveries in India [35,37]. Further qualitative studies should be conducted using multiple methods, such as in-depth interviews with women and TBAs, to understand care-seeking behavior and why women with fewer pregnancies are more likely to seek treatment from UAPs.

Results of recent studies have shown that UAPs were called for management of delivery-related complications in a rural area of Bangladesh [12,38]. The findings of our study also revealed that 45.2% of women who reported at least one complication sought care from a UAP. In contrast, the 2007 Bangladesh Demographic and Health Survey (BDHS) found that unqualified doctors assisted only 23% of women who reported at least one delivery-related complication, although the BDHS did not present these findings disaggregated by area (urban or rural) [39].

The UAP's practice during the third stage of labor seems to be beneficial. Most UAPs reported that they referred women with retained placenta directly to a health facility, although some UAPs did report providing oxytocin to expel the placenta. This finding is similar to another study in India where 2% of women with home births reported that village practitioners provided oxytocin during the third stage of labor [40]. However, WHO currently recommends that oxytocin during the third stage be administered by skilled providers [41].

The findings of in-depth interviews showed that the UAPs commonly used different types of medicines (anaroxyl and methergine) in both tablet and injectable forms to manage excessive bleeding just after childbirth (after both baby and placenta were delivered). This practice should be monitored because the improper use of medicines can be harmful. For example, intravenous methergine injection requires close monitoring by a qualified doctor to avoid sudden increases in blood pressure or stroke [41,42].

During the in-depth interviews, the UAPs disclosed referring women to health facilities for various health problems. This finding corroborates Parkhurst and Rahman's findings from a qualitative study [12] but differs from our quantitative findings which showed that UAPs referred only 6% of women (9 of 149) to health facilities. This discrepancy may be because of different study designs. For example, Parkhurst and Rahman interviewed only women who were referred to a health a facility while our study included all women with a trial of labor at home. Moreover, UAPs might have some knowledge on referral care but they may not be doing in practice (response bias). Further research on knowledge, attitude, and practice of UAPs regarding maternal healthcare may help to understand the reasons behind this discrepancy.

One limitation of this study was that the findings were based on only one slum area (Kamrangir Char slum). A further study should be conducted in other slums of Dhaka and outside Dhaka for generalization. We also do not know if knowledge of pregnancy danger-signs of women developed before or after delivery. To minimize the possible recall bias, we tried to limit the recall period up to six months. Through qualitative interviews, we explored the UAPs' practices based on their own responses, and they therefore may not fully represent harmful practices.

Based on the findings of the present study, several recommendations can be made. The Manoshi project and other maternal and newborn health programs in urban areas should target slum women as in India and Nepal [43,44], especially those who have fewer pregnancies in their lifetime, to motivate them to seek healthcare from health facilities rather than from UAPs. Health-education messages can also be used to help women understand where to seek care for delivery-related complications. We found that one-third of the women received services from the UAPs. UAPs are often respected members of communities, [12] and could be used as change agents by local health programs to encourage referral to health facilities. UAPs should also be educated and trained on harmful effects of oxytocin and other medications. Maternal and newborn health programs may introduce a guideline for the management of postpartum hemorrhage for UAPs and advocate that UAPs refer women to skilled providers if there is excessive bleeding during the immediate postpartum. Finally, UAPs are increasing and have formed their own professional association in Bangladesh [20]. This association should be a key partner on health programs to prevent harmful practices.

## Conclusion

This study has demonstrated that one-third of the women sought care from the UAPs during labor and delivery in the urban slums of Dhaka. The delivery care practices of UAPs can be harmful, which could result in adverse outcomes for both woman and baby. It is necessary to develop and test effective interventions to improve health care-seeking from skilled providers to ensure that women receive appropriate perinatal care.

## Competing interests

The authors declare that they have no competing interests.

## Authors' contributions

TW assisted the principle investigator (PI) in designing and implementing the study, coordination, data analysis and wrote the manuscript. ACM was the PI of the study, involved actively in its design, implementation, data analysis, revising the manuscript. MI supervised TW in writing manuscript on behalf of PI. TW, ACM and MI read and approved the final manuscript.

## Pre-publication history

The pre-publication history for this paper can be accessed here:

http://www.biomedcentral.com/1471-2393/10/50/prepub
